# Does Methylphenidate Work in Children and Adolescents with Attention Deficit Hyperactivity Disorder?

**DOI:** 10.3390/pediatric13030050

**Published:** 2021-08-01

**Authors:** Johanne Pereira Ribeiro, Emma Jasmine Arthur, Christian Gluud, Erik Simonsen, Ole Jakob Storebø

**Affiliations:** 1 Center for Evidence-Based Psychiatry, Psychiatric Research Unit, Psychiatry Region Zealand, 4200 Slagelse, Denmark; joper@regionsjaelland.dk (J.P.R.); emma.jaa.live.no@gmail.com (E.J.A.); es@regionsjaelland.dk (E.S.); 2 Department of Regional Health Research, Faculty of Health Sciences, University of Southern Denmark, 5000 Odense, Denmark; cgluud@ctu.dk; 3 The Copenhagen Trial Unit, Centre for Clinical Intervention Research, The Capital Region, Copenhagen University Hospital, Rigshospitalet, 2100 Copenhagen, Denmark; 4 Department of Clinical Medicine, University of Copenhagen, 2100 Copenhagen, Denmark; 5 Department of Child and Adolescent Psychiatry, Psychiatry Region Zealand, 4000 Roskilde, Denmark; 6 Department of Psychology, University of Southern Denmark, 5000 Odense, Denmark

**Keywords:** ADHD, attention deficit hyperactivity disorder, methylphenidate, stimulants, children, adolescents

## Abstract

Objective: Attention deficit hyperactivity disorder (ADHD) is a common psychiatric disorder diagnosed in increasing proportions of children and adolescents. The psychostimulant methylphenidate has been considered the first-line pharmacological treatment for children and adolescents with ADHD for more than 60 years. Considering recent publications on methylphenidate for ADHD, we here give an overview of its effects in children and adolescents with ADHD, elicited by a well-disputed Cochrane review and narratively synthesise the evidence in the field. Method: We searched for systematic reviews and meta-analyses that investigated methylphenidate as an intervention for children and adolescence with ADHD compared with placebo or no treatment. We assessed the quality of the evidence using AMSTAR II. Results: We found 24 eligible systematic reviews and meta-analyses of which 11 were rated as high- quality evidence according to AMSTAR II. The evidence claiming that methylphenidate is beneficial in treating children and adolescents with ADHD was of very low certainty. The underreporting of adverse events in randomised clinical trials may impede an adequate depiction of the balance between benefits and harms. Conclusions: It appears that there is uncertain evidence on group-level to support the claim that methylphenidate is beneficial in treating children and adolescents with ADHD. Future randomised clinical trials and systematic reviews should include individual participant data, which would allow us to assess intervention effects across modifiers, like age, sex, ADHD subtypes, comorbidities, and dose.

## 1. Introduction

The attention deficit hyperactivity disorder (ADHD) diagnosis is based on persistent impairment of one or more of the following core symptoms: excessive inattention, hyperactivity, and impulsivity. Furthermore, symptoms must interfere with everyday life before the age of 7 years (ICD-10) or 12 years (DSM-5) [1,2]. Children and adolescents with ADHD are at increased risk of a broad spectrum of co-occurring conditions [3,4]. The disorder is diagnosed in increasing proportions of children and adolescents, which brings focus to the central nervous stimulant methylphenidate. This drug has been considered the first-line pharmacological treatment for children and adolescents with ADHD for more than 60 years [5].

The 2015 Cochrane review by Storebø and colleagues investigated the beneficial and harmful effects of methylphenidate as treatment for children and adolescents with ADHD [6,7]. Storebø and colleagues included 185 randomised clinical trials and found small potential beneficial effects of methylphenidate versus placebo or no-intervention. However, they noted that there are mutable, methodological flaws, such as lack of blinding and outcome reporting bias in the included trials, which prevented a clear estimation on the magnitude of intervention effects [6,7]. Storebø and colleagues found that methylphenidate may improve teacher-rated ADHD symptoms (standardised mean difference (SMD) −0.77, 95% confidence interval (CI) −0.90 to −0.64; 19 trials, 1698 participants). The evidence was rated as very low-quality evidence due to risk of bias and heterogeneity. Teacher-rated general behaviour seemed to improve with methylphenidate (SMD −0.87, 95% CI −1.04 to −0.71; 5 trials, 668 participants). The evidence was rated as very low-quality evidence due to risk of bias and indirectness.

There was a small beneficial effect of methylphenidate on quality of life (SMD 0.61, 95% CI 0.42 to 0.80, 3 trials, 514 participants). The evidence was rated as of very low-quality due to high risk of bias, indirectness, and selective outcome reporting [7].

Methylphenidate significantly increased adverse events considered non-serious and the risks of serious adverse events could not be assessed due to lack of data [7]. This review was heavily criticised in articles and editorials [8,9,10,11,12,13]. The criticism was focused on erratic inclusion of studies, risk of bias assessment, and assessment of the quality of included studies and meta-analyses as well as errors in the data. The criticism was rebutted and it was shown that the evidence base for the use of methylphenidate in children and adolescents is in fact flawed [14,15,16,17,18,19]. None of the critical comments that led to amendments changed anything major in the results or conclusions. This viewpoint provides an overview of the evidence since the 2015 Cochrane review by Storebø and colleagues [6,7]. In this article, we consider the effects of methylphenidate in children and adolescents with ADHD and comorbid disorders compared with placebo or no-interventions.

## 2. What New Publications Are out There?

We searched PubMed, the Cochrane Library, and BMJ Best Practice on 6 October 2020, and achieved 477 full text hits after removing duplicates and irrelevant publications. Two independent reviewers went through titles and abstracts of these and excluded 449 studies, leaving 24 included studies. A third reviewer resolved disagreements. In cases where any author of this article was also co-author in an included review, we excluded that co-author from the assessment process. We included 24 reviews or meta-analyses in English, published in 2013 and onwards that investigated methylphenidate as an intervention for children and adolescence with ADHD compared with placebo or no treatment. Reviews investigating multiple interventions were included if data on methylphenidate was reported separately and it was possible to differentiate from other interventions. Two reviewers using the AMSTAR II checklist assessed the 24 included studies [20]. Of these, 11 reviews were of high quality (see Table 1) [21,22,23,24,25,26,27,28,29,30,31]. The remaining studies albeit relevant and fulfilling inclusion criteria had some concerns that affected the overall certainty of evidence. Some had not published a protocol prior to their study [32,33,34,35,36,37,38], had no comprehensive literature search [33,35,39], were unclear on whether study inclusion and data extraction were performed in duplicates [33,40], had not conducted a thorough quality assessment [32,34,35,38,41,42], had no report on risk of bias assessment of included studies [33,34,35,38,40,41,42,43,44], or had a combination of two or more of these caveats.

## 3. Assessment of Methylphenidate by Expert Groups

The updated 2018 guideline from the National Institute for Health and Care Excellence (NICE) recommends methylphenidate as the first-line pharmacological treatment for children over 5 and adolescents [45]. There are several methodological problems with the NICE guideline on ADHD, regarding erratic assessment of the certainty of the included studies and selection bias as the authors only included 16 trials on methylphenidate in children and adolescents [46]. These limitations in the NICE guideline raises concerns about the validity of the recommendations.

Two researchers from Mount Sinai Graduate Program in Public Health applied for inclusion of methylphenidate on the WHO Model List of Essential Medicines in December 2018 for children, adolescents, and adults with ADHD [47]. The WHO Expert Committees rejected the application due to concerns regarding the quality and interpretation of the evidence for benefits and harms [48,49]. The decision was unanimous [48,49].

## 4. How Efficient Is Methylphenidate in Recent Reviews or Meta-Analyses?

Using individual participant data from 39 patients in n-of 1-trials in their meta-analysis, Punja and colleagues. (2016) concluded methylphenidate to be effective for ADHD in children [28]. The generalisability of the results however, may be limited as data from only one study contributed to most of the analyses. Catala-Lopez and colleagues (2017) included 40 trials on methylphenidate containing 3836 participants [21]. Catala-Lopez and colleagues deemed methylphenidate an efficient treatment for ADHD symptoms especially when combined with behavioural therapy. This conclusion was made with low to very low certainty in the evidence [21].

Utilising methylphenidate as the pharmacological first choice of treatment for children and adolescents with ADHD was supported in a large systematic review and network meta-analysis by Cortese and colleagues (2018), who compared efficacy and tolerability of methylphenidate for ADHD to placebo alongside other medications [23]. The authors however excluded many relevant trials to fulfil statistical and methodological assumptions in the network meta-analyses and thereby increased the risk of selection bias [50]. They did this because they believed that including these trials would have been a clear violation to their published protocol and also that this would have compromised the transitivity of the network meta-analyses [51]. Furthermore, the authors assessed all the indirect comparisons at low to very low quality of evidence. Indirect evidence differentiates network meta-analyses from conventional meta-analyses, and given the decreased interpretability of these data, the overall findings might not be more comprehensive or novel than previous reviews.

In an efficacy and safety assessment of stimulants and non-stimulants on ADHD symptoms for children and adolescents, Cerrillo-Urbina and colleagues (2018) concluded that both treatments alleviate ADHD symptoms and that stimulants especially seemed to have a superior effect on inattention and hyperactivity. Their review included only four trials on methylphenidate which were all conducted before 2013 and therefore did not encompass any new evidence [22].

Incorporating comorbidity in an effect analysis, a Cochrane review by Osland and colleagues (2018) investigated the effects of various pharmacological treatments, including methylphenidate for ADHD in children with comorbid tic disorder [27]. They found improvements of symptoms of ADHD and tics using methylphenidate based on low-certainty evidence.

## 5. What Are the Harms of Methylphenidate?

When assessing the effects of methylphenidate, importance should be given to the balance between benefits as well as adverse effects.

Adverse effects of methylphenidate commonly investigated and reported include inter alia insomnia, anxiety, growth suppression, reduced weight gain and gastrointestinal events like nausea, abdominal pain, and reduced appetite [7]. Osland and colleagues. found that methylphenidate was associated with appetite suppression, weight loss, and insomnia [27].

The 2018 Cochrane systematic review of non-randomised studies on methylphenidate and adverse events in children and adolescents with ADHD by Storebø and colleagues, showed that methylphenidate may be associated with a number of serious adverse events as well as a large number of non-serious adverse events in children and adolescents, which often lead to withdrawal of methylphenidate [31]. The certainty in the evidence was very low, and accordingly, it was not possible to accurately estimate the risk of adverse events [31], which is likely higher than reported [31,52,53]. The 2015 Cochrane review by Storebø and colleagues [7] associated the use of short-term methylphenidate for ADHD in children and adolescents with a risk increase of appetite decline, weight loss, and abdominal pain [24]. Adverse effects require close monitoring, while clinical experiences suggest that most non-serious adverse effects disappear within a couple of weeks. Further, Storebø and colleagues [7] investigated the occurrence of psychotic symptoms in children and adolescents with ADHD receiving methylphenidate [29]. It was not possible to conclude on this matter due to low quality evidence and sparse data from an otherwise rigorous and exhaustive gathering of evidence [29].

When comparing treatment with methylphenidate to non-pharmacologic interventions, Kemper and colleagues found that methylphenidate increased sleep disturbance and decreased appetite. All but two included studies, however, were rated as having insufficient certainty of evidence by the authors, for which reason they decided that no other outcomes could be investigated [25].

Other serious adverse events of methylphenidate treatment for children and adolescents include the development of psychosis and cardiovascular problems. Investigating cardiovascular risks of methylphenidate and other pharmacological treatments for ADHD, Liu and colleagues could not exclude a modest elevated risk for sudden cardiac arrest and arrhythmia linked to the use of methylphenidate, based on confidence intervals. They advised that this risk be considered against the benefits of medication and suggested regular monitoring of heart rate and blood pressure [26].

## 6. Balance between Benefits and Harms

The underreporting of adverse events in randomised clinical trials may impede an adequate depiction of the balance between methylphenidate benefits and harms in children and adolescents with ADHD. The issue of underreporting adverse events is well known and has been investigated repeatedly [54,55]. In a recent umbrella review, Solmi and colleagues investigated adverse effects of pharmacological treatments for ADHD in children and adolescents [30]. According to Solmi and colleagues, only 32.1 percent of the literature on methylphenidate (which was the highest coverage in the group of ADHD medications) covered adverse events. In this group, only 6.4 percent found these to be significantly worse with methylphenidate compared to placebo. They found that participants in the methylphenidate group were much more likely to experience insomnia compared with participants in the placebo group (odds ratio: 4.7; 95% CI 1.9–10.9). They also found an increased relative risk of gastrointestinal adverse events: abdominal pain (relative risk (RR) 1.5; 95% CI 1.3–1.8), anorexia (RR 3.2; 95% CI 2.6–3.9), and nausea/vomiting (RR 1.4; 95% CI 1.0–1.8). Additionally, the standardised mean difference for weight loss was −0.77 with 95% CI −1.1 to −0.5, in the methylphenidate group compared with the placebo group [30].

None of the included reviews of good quality from our search mentioned the effects of withdrawal symptoms. It is important to investigate collateral cognitive and emotional withdrawal effects such as s depression, poor concentration, agitation, irritability, and anxiety [56].

Consequently, when comparing benefits and harms it seems there are substantial risks of harms, which may have been overlooked in previous publications [57]. Against these risks, one has to evaluate the benefits. Very low certainty evidence suggests limited benefits of methylphenidate based on aggregate data [7,21,22,23,27]. Furthermore, the benefits of methylphenidate are solely assessed in trials with less than a 3-month duration [7,23]. We lack evidence supporting long-term treatments, as is the usual way of using the medication [7,23].

We cannot of course ignore that some patients and clinicians experience advantages of methylphenidate. When looking at the evidence from clinical trials, the variance of the outcomes in the methylphenidate group does not seem to be larger than that of the placebo or no-intervention group. This makes it unclear whether there might be a differential effect between the groups [58]. Additionally, it is worth mentioning that the case is quite similar if we assess the effects of methylphenidate in adults with ADHD. That is, likely more harms than benefits. A new network meta-analysis investigating the benefits and harms of pharmacological treatment for adults showed possible beneficial effect of methylphenidate compared with placebo, however, certainty of the evidence was low to very low and there was limited reporting on long-term adverse events [59]. Recent systematic reviews question the effects of methylphenidate for adults with ADHD [59,60,61].

## 7. Whom to Treat?

Many experienced clinicians and researchers find that methylphenidate gives symptom reduction in some children and adolescents with ADHD, albeit the proportion of this group is unclear. Although the average treatment effect may not be above the clinically significant level, beneficial effects in one or more subgroups can occur. One way to identify such subgroups is to conduct individual patient data (IPD) reviews. In this type of review it is possible to look for information on whether patient characteristics influence treatment outcomes [62]. Moreover, potential symptom reduction in undefined subgroups should always be weighed against both serious and non-serious adverse events. The true number of adverse events remains uncertain given the insufficient quality of evidence and poor documentation of harms. Therefore, methylphenidate might potentially cause more harm for children and adolescents with ADHD than our studies indicate [17]. Swanepoel and colleagues published an article that focused on evolutionary thinking and how it can help to understand ADHD [63]. They argued that some children with ADHD struggle with the expectations in school to do sedentary work and concentrate for hours. This environment may, for some children, be the reason for their inattention and hyperactivity symptoms. This may be accommodated by a change in the environment, e.g., more physical activity in school [63] and potentially mindfulness-based therapies [64]. For the children where clinicians decide that medication is necessary, it may be important to evaluate benefits and harms with a systematic approach. Such an approach could take into account comorbidities, cardiovascular irregularities, sleeping and eating patterns, genetic heredity, and other risk factors. Potential benefits and harms should be discussed with patients and their caregivers [17]. An appropriate monitoring system that closely assesses the treatment benefits and harms over time should be used [17]. Careful titration is required to determine optimal dose and length of treatment. Medication should be discontinued if no improvement has occurred and a structured treatment-interruption should be considered due to unclear evidence and risk of harmful effects.

## 8. Conclusions

It appears that there may still be uncertain evidence on group-level to support the conclusion that methylphenidate would be beneficial in treating children and adolescents with ADHD. Future randomised clinical trials and systematic reviews should include individual participant data, which would allow us to assess intervention effects across modifiers, like age, sex, ADHD subtypes, comorbidities, and dose. These data must be present for both the short- and long-term effects of methylphenidate for children and adolescents. Only then, can we discover the subgroups of patients with ADHD that benefit the most from methylphenidate, as well as those that benefit the least.

## Figures and Tables

**Table 1:** Quality assessment of included studies.

Study	Number of Included Trials or Studies	Comorbidity	Adverse Events	PICO §	Quality Assessment	Protocol	Comprehensive Literature Search	Duplicate Study Selection	Duplicate Data Extraction	List of Excluded Studies and Justification	Included Studies Described Adequately	Risk of Bias Assessment	Discussion of Heterogeneity	Investigation of Publication Bias and Discussion of Impact on Results	Sources of Funding for Included Studies Reported	Appropriate Methods for Statistical Combination of Results in Meta-Analysis	Impact of RoB in Individual Studies on the Results of the Meta-Analysis or Other Evidence Synthesis Assessed	RoB in Individual Studies Accounted for in Interpretation/Discussion of Results	Authors Funding Sources and Conflicts of Interest Described
**Catala-Lopez 2017**	**190** **(n = 26,114)**	**Yes**	**Yes**	**4/4**	**Yes**	**Yes**	**Yes**	**Yes**	**Yes**	**No**	**Yes**	**Yes**	**Yes**	**Yes**	**Yes**	**Yes**	**Yes**	**Yes**	**Yes**
**Cerrillo-Urbina 2018**	**15** **(n = 4248)**	**Unclear**	**Yes**	**4/4**	**Yes**	**Yes**	**Yes**	**Yes**	**Yes**	**No**	**Partially yes**	**Yes**	**Yes**	**Yes**	**No**	**Yes**	**Yes**	**Yes**	**Yes**
**Holmskov 2017**	**185** **(n = 12,245)**	**Yes**	**Yes**	**4/4**	**Yes**	**Yes**	**Yes**	**Yes**	**Yes**	**Yes**	**Yes**	**Yes**	**Yes**	**Yes**	**Yes**	**Yes**	**Yes**	**Yes**	**Yes**
**Kemper 2018**	**90** **(n = unclear)**	**Yes**	**Yes**	**4/4**	**Yes**	**Yes**	**Yes**	**Yes**	**Yes**	**Yes**	**Yes**	**Yes**	**Yes**	**Yes**	**Yes**	**Yes**	**Yes**	**Yes**	**Yes**
**Cortese 2018**	**133** **(n = 24,642)**	**Yes**	**Yes**	**4/4**	**Yes**	**Yes**	**Yes**	**Unclear**	**Yes**	**Yes**	**Yes**	**Yes**	**Yes**	**Yes**	**Yes**	**Yes**	**Yes**	**Yes**	**Yes**
**Liu 2019**	10 ** **(n = 4,221,929)**	**Yes**	**Yes**	**3/4**	**Yes**	**No**	**Yes**	**Yes**	**Yes**	**No**	**Yes**	**Yes**	**Yes**	**Yes**	**No**	**Yes**	**No**	**No**	**Yes**
**Ramstad 2018**	**27** **(n = 76,237)**	**No**	**Yes**	**4/4**	**Yes**	**Yes**	**Yes**	**Yes**	**Yes**	**No**	**Yes**	**Yes**	**Yes**	**Yes**	**Yes**	**Yes**	**Yes**	**Yes**	**Yes**
**Storebø 2018**	260 ** **(n = 2,283,509)**	**Yes**	**Yes**	**4/4**	**Yes**	**Yes**	**Yes**	**Yes**	**Yes**	**Yes**	**Yes**	**Yes**	**Yes**	**Yes**	**Yes**	**Yes**	**Yes**	**Yes**	**Yes**
**Solmi 2020**	138 *** **(n = 336,065)**	**Unclear**	**Yes**	**4/4**	**Yes**	**Yes**	**Yes**	**Yes**	**Yes**	**No**	**No**	**Yes**	**No**	**No**	**No**	**No meta-analysis**	**No meta-analysis**	**No meta-analysis**	**Yes**
**Osland 2018**	**8** **(n = 510)**	**Yes**	**Unclear**	**4/4**	**Yes**	**Yes**	**Yes**	**Yes**	**Yes**	**Yes**	**Yes**	**Yes**	**Yes**	**No**	**Yes**	**No meta-analysis**	**No meta-analysis**	**No meta-analysis**	**No**
**Punja 2016**	3 * **(n = 39)**	**Unclear**	**Yes**	**4/4**	**Partially yes**	**No**	**Yes**	**Yes**	**Yes**	**No**	**Yes**	**Yes**	**Yes**	**Yes**	**No**	**Yes**	**Yes**	**Yes**	**Yes**
Coghill 2014	60 (n = unclear)	Yes	No	3/4	No	Yes	Yes	Yes	Yes	Yes	No	No	Yes	No	No	Yes	No	No	Yes
Stuckelman 2017	32 (n = 3664)	No	No	4/4	No	No	No	Yes	Yes	No	No	No	Yes	Yes	No	Yes	No	No	Yes
Hennissen 2017	18 (n = 5837)	Yes	Yes	4/4	No	No	Yes	Yes	No	No	Yes	No	Yes	No	No	Yes	No	No	Yes
Joseph 2017	36 (n = unclear)	Yes	Yes	4/4	Yes	Yes	Yes	Yes	Yes	No	No	No	Yes	No	No	Yes	No	No	Yes
Kambeitz 2014	16 (n = 1572)	Unclear	No	3/4	No	No	No	No	No	No	Yes	No	Yes	Yes	No	Yes	No	No	Yes
Li 2017	62 (n = 12,930)	Unclear	Yes	4/4	No	No	Yes	Yes	Yes	No	Yes	No	Yes	No	No	Yes	No	No	No
Liang 2018	22 (n = 46,107)	Unclear	Yes	3/4	Yes	Yes	Yes	Yes	Yes	No	Yes	No	Yes	Yes	No	Yes	No	No	Yes
Maia 2017	7 (n = 444)	Unclear	Yes	3/4	Yes	No	Yes	Yes	Yes	Yes	Yes	No	Yes	No	Yes	Yes	No	No	Yes
Myer 2018	36 (n = 3647)	No	Yes	2/4	Yes	No	No	Yes	No	No	Yes	No	Yes	Yes	No	Yes	No	No	Yes
Pozzi 2018	45 (n = unclear)	Yes	Yes	3/4	No	No	No	Yes	Yes	No	Yes	Unclear	Yes	Yes	No	Yes	No	No	Yes
Prasad 2013	43 (n = 2110)	No	No	4/4	Yes	No	Yes	Yes	Yes	No	Yes	Yes	Yes	Yes	No	Yes	No	Yes	Yes
Punja 2013	13 (n = 882)	No	Yes	4/4	No	No	Yes	Yes	Yes	No	Yes	Yes	Yes	No	Yes	Yes	Yes	No	Yes
Rezaei 2016	11 (n = 2772)	Yes	Unclear	4/4	Yes	No	Yes	Yes	Yes	No	No	No	Yes	Yes	No	Yes	No	No	Yes

Studies with bold caption rated as good quality evidence according to AMSTAR 2. § Participants, intervention, control, outcomes. * Individual participant data (IPD). ** Non-randomized studies of intervention effects (NRSI). *** Combined randomized clinical trials and NRSI.
